# Supplementary material for: Cancer Screening among Rural People Who Use Drugs: Colliding Risks and Barriers
Source: Int J Environ Res Public Health. 2022 Apr 10;19(8):4555. doi: 10.3390/ijerph19084555 (PMC9026855; doi:10.3390/ijerph19084555)
Supplement: Supplementary file 1 [file ijerph-19-04555-s001.zip › ijerph-1629893-supplementary.pdf]

SURVEY INSTRUMENT

A. Demographic Information

1. What is your age?

2. What is your gender (at birth)?
   Male Female Other

3. What is your sexual orientation/ gender identity? Do you consider yourself to be:
   a. Heterosexual/Straight
   b. Lesbian or gay
   c. Bisexual
   d. Other
   e. Don’t know/ Not sure

4. Which of the following would you self-identify as your race?
   a. Black or African American
   b. White
   c. Hispanic
   d. Asian
   e. Other

5. What county are you from?

6. What is your current relationship status?
   a. Never married
   b. Divorced
   c. Widowed
   d. Separated
   e. Married
   f. Co-habiting/living together

7. What is your household income?
   a. Less than $25,000
   b. $25,000 to $34,999
   c. $35,000 to $49,999
   d. $50,000 to $74,999
   e. $75,000 to $99,999
   f. $100,000 or more

8. What is the highest grade or year of school you completed?

   a. Never attended school or only kindergarten
   b. Elementary
   c. Some high school

d. High school graduate
e. Some college or technical school
f. College graduate or more

9. How much do you weigh without shoes?
   _ _ _ lbs

10. How tall are you without shoes?
   _ _ _ ft _ _ _in

11. During the past 30 days, on the days when you drank, about how many drinks did you drink on the average?
   _ _ _ Number of alcoholic beverage

12. During the past 30 days, how many days per week did you have at least one drink of any alcoholic beverage?
   _ _ _ Number of days

13. In a typical week, how many days do you do any physical activity or exercise of at least moderate intensity?
   a. None
   b. _ _ _ Number of days per week
   c. Don't know/Not sure

14. During the past month, did you participate in any physical activities or exercises such as running, yoga, golf, gardening, or walking for exercise?
   a. No
   b. Yes
   c. Don't know/not sure

15. Do you now, or have you in the past, smoked cigarettes?
   Yes No

16. If 'yes', for how many years did you/have you smoked?

B. <u>How Drug Use Might Have Impacted Your Medical Care</u>

17. Do you currently see a <u>primary care</u> provider on a regular or routine basis? (This might be for annual exams, flu vaccinations, etc.)?

   Yes No

18. If you have a primary care provider, how long have you been going to this provider? (select only one response)

a. Less than 6 months
b. At least 6 months but less than 1 year
c. At least 1 year but less than 3 years
d. At least 3 years but less than 5 years
e. 5 years or more

19. Have you ever avoided health care because of your drug use?
    Yes No

20. Have you ever had a healthcare worker refuse to treat you or denied you access to medical treatment or care because of your drug use?
    Yes No N/A – never accessed healthcare

21. Do you feel 'accepted' and "non-judged" during office visits?
    a. Yes, and they know about the drug use
    b. Yes, but they don't know about the drug use
    c. No, they treat me differently

22. Have you had bad experiences with primary care such that you considered not going anymore?
    Yes No

C. Questions about Cancer Screening Knowledge

Screening refers to quick tests to see if you might have a specific disease, such as cancer. Unfortunately, there are only a couple cancers for which there is a good screening test.

23. Please check if you think there is, or is not, a screening test for each of the following cancers.

	Yes	No
- breast	___	___
- stomach	___	___
- cervical	___	___
- brain	___	___
- liver	___	___
- lung	___	___
- kidney	___	___
- colorectal	___	___
- eye	___	___

D. Questions about Cancer Screening Recommendations

8. Has a doctor, nurse, or other health professional ever recommended that you be screened for breast cancer by any of the following?

	Yes	No
- mammography	___	___
- breast MRI	___	___

9. Has a doctor, nurse, or other health professional ever recommended that you be screened for cervical cancer by any of the following?

	Yes	No
- Pap test	___	___
- HPV DNA	___	___

10. Has a doctor, nurse, or other health professional ever recommended that you be screened for colorectal cancer by any of the following?

	Yes	No
- colonoscopy	___	___
- virtual colonoscopy (CT colonography)	___	___
- sigmoidoscopy	___	___
- stool (fecal) tests	___	___

11. Has a doctor, nurse, or other health professional ever recommended that you be screened for lung cancer?

	Yes	No
- Low dose computed tomography (LDCT)	___	___

E. Questions about Cancer Screening Utilization

12. Have you received any of these screening tests in the past 10 years (Yes/No). If 'Yes", please check which years for each.

Location	Screening type	Ever Screened?		Which years? (from 2011 on)									
		No	Yes	11	12	13	14	15	16	17	18	19	20
breast	mammography												
	breast MRI												
cervix	Pap test												
	HPV DNA												
colon and rectum	colonoscopy												
	virtual colonoscopy (CT colonography)												
	Sigmoidoscopy												
	stool (fecal) tests												
lung	Low dose computed tomography (LDCT)												

13. There are many reasons why people do not receive cancer screening. If cancer screening was ever recommended to you in the past and you did not actually receive the screening, please indicate all the reasons why. *Please check all that apply.*

___ I didn't have insurance
___ I didn't think I could afford it
___ I didn't have a way to get to the screening location (no transportation)
___ I couldn't get an appointment
___ I couldn't find the right doctor
___ I was embarrassed to discuss or go through with it
___ I didn't want to know

___ I didn't think I was at risk
___ I didn't have any symptoms
___ I don't have a family history
___ I thought the test might be difficult
___ I thought the test might be painful
___ I was afraid I'd be judged or disrespected during the visit

F. <u>Questions about Cancer History</u>

14. Have you ever told from health care providers that you have a cancer?

Yes No Don't know / Not sure

15. Has a parent, sibling or child related to you by blood ever been diagnosed with cancer? *Include only siblings with same biological mother and father.*

Yes No Don't know / Not sure

INTERVIEW GUIDE

PWUD/Cancer Screening Preliminary Interviews – PWUD

Note to Interviewer: After going through informed consent process, provide participant with incentive.

After consent, turn on the recorder and say:

"Today is [date]. This is an interview with [interview code number]. Before we start the interview, I'd like to say that I am recording you, and ask if you give me your permission to record you."

Introduction

Interviewer Script: I want to thank you for talking with me today. We are interested in learning more about what people in the area know about cancer screenings and adherence to cancer screening recommendations. I want to remind you that everything you say is confidential. We will not share your name with anyone other than study staff.

This interview is completely voluntary. If you are uncomfortable with any question, and do not want to answer, please let me know and we can skip it.

Do you have any questions before we begin?

[Take time to answer questions if necessary.]

Interviewer Script: I'd like to start by asking you about cancer screening guidelines. Cancer screening increases the chances of detecting certain cancers early, when they might be easier to treat. Screening guidelines help us know when to start screening and how often to do screening. Recommendations vary by age, sex, etc.

1. Can you tell me what you know about the types of cancer screenings you should do?
    a. Probe: When should you start colonoscopy, mammography, pap test, etc.? (list gender-specific screenings)
    b. Probe: How often do you need to get colonoscopy, mammography, pap test, etc.? (list gender-specific screenings)
    c. Prompt: How/where did you learn about these guidelines?
    d. Prompt: What about for other people? What types of screening should they get and when?
2. Have you ever been told that you needed a certain screening test?
    a. Prompt: Colonoscopy, mammogram, pap smear, colonoscopy, stool (fecal) test, low-dose computed tomography (LDCT).

    b. If yes to any: What happened? Did you go for the screening? What was your experience doing this?
    c. If no to any (gender-specific): Why do you think your providers haven't told you to get screening?
3. Can you tell me about any barriers you face getting recommended cancer screenings?
    a. Probe: What has been your experience getting medical care?
        i. Probe: Does drug use come up?
        ii. Probe for stigma.
    b. Probe: Do you have insurance?
    c. Probe: If you had a medical appointment, how would you get there?
    d. Probe: How often do you have appointments that are scheduled a few weeks or months away? Is it easy for you to make it to these appointments?
4. What would make you feel more supported and better able to access recommended cancer screenings?
    a. Probe: Standard office visits vs. mobile/off-clinic visits.
